# Anesthetic management of a patient with prosthetic heart valve for non-cardiac surgery: A case report

**DOI:** 10.1186/1757-1626-1-196

**Published:** 2008-09-30

**Authors:** Goneppanavar Umesh, Swati Verma, Kaur Jasvinder

**Affiliations:** 1 Department of Anaesthesiology, Kasturba Medical College, Manipal, India

## Abstract

**Background:**

Patients with prosthetic heart valves are a challenge to any anesthesiologist due to the risk of infective endocarditis, bleeding and thrombosis.

**Case presentation:**

We present anesthetic management of a 58-year-old Indian lady with a prosthetic (mechanical) heart valve who underwent hemireplacement arthroplasty.

**Conclusion:**

Patients with prosthetic heart valves, especially those with the mechanical valves are prone for thrombosis and resultant complications if anticoagulation is not maintained properly. However, when they are scheduled for major surgery, they can be best managed by normalising the coagulation profile immediately prior to surgery and restarting the anticoagulation as early as possible.

## Background

Management of patients with prosthetic heart valves for non-cardiac surgery involves cardiac assessment for valvular function, residual pathology, infective endocarditis and functional status; assessment of the status of anticoagulation, any risk of bleeding, preparation for reversal of anticoagulants if needed intraoperatively; and neurological evaluation for detecting any impairment due to thromboembolism. We present successful anesthetic management of a lady with prosthetic mitral and aortic valves who underwent hemireplacement arthroplasty of her right hip.

## Case presentation

A 58-year-old Indian lady, a known case of rheumatic heart disease since the age of 16 years who had undergone a successful mitral and aortic valve replacement surgery 11 years previously, sustained a fall and fractured the neck of her right femur. She was scheduled for hemireplacement arthroplasty. She had not sustained any head injury during the fall. She was a known diabetic on insulin therapy. Her past history revealed that prior to the valve replacement, she used to get breathless even while doing routine daily activities. Presently, she could climb two flights of stairs without any syncope, palpitations, fatigue, chest pain or breathlessness prior to the trauma. She was receiving oral warfarin 2 mg once daily.

**Figure 1:**
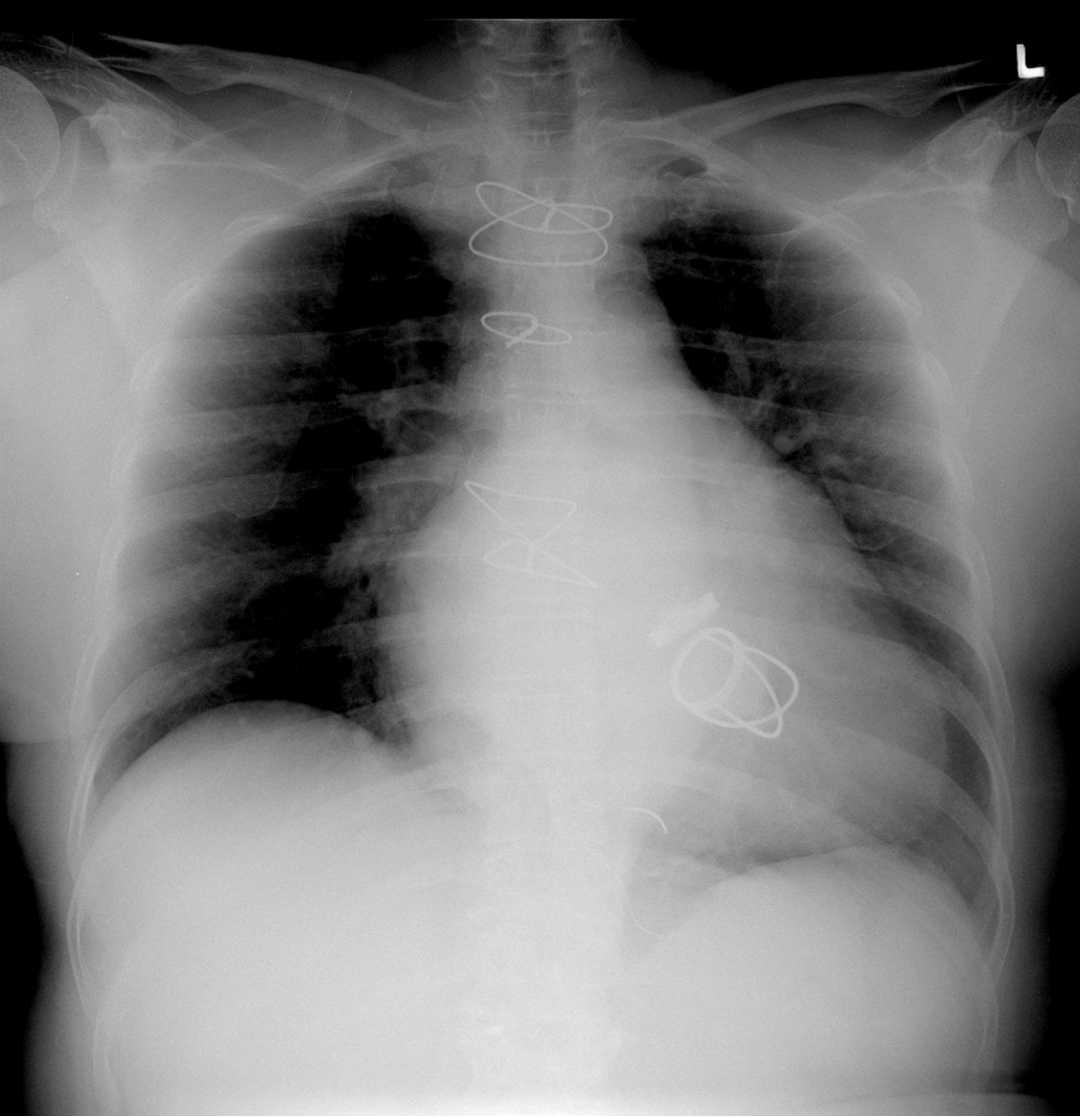
CXR showing cardiomegaly with ball in cage mechanical prosthesis.

Physical examination revealed her to be afebrile, with an irregularly irregular pulse-104 beats per minute, respiratory rate 18 breaths per minute and blood pressure 160/86 mmHg. Her systemic examination did not reveal any pathology. Patient's electrocardiogram showed atrial fibrillation with a heart rate of 110 per minute. Her ECHO showed 54% ejection fraction, normally functioning valves, no paravalvular leak and confirmed absence of vegetations and clots. The chest X ray revealed ball in cage mitral valve prosthesis, an aortic prosthesis, sternotomy sutures and cardiomegaly (Fig 1). Her preoperative laboratory evaluation reports are given in Table 1.

**Table 1:** Laboratory evaluation reports

Hemoglobin	9.3 g/dL
Hematocrit	27.3

Total leucocyte count, differential count	6500, neutrophils 60%, lymphocytes 28%, monocytes 7%, eosinophils 4%, basophils 1%

Fasting blood sugar	103 mg/dL

Blood urea	28 mg/dL

Serum creatinine	0.9 mg/dL

Serum electrolytes	Na^+ ^139 mEq/L, K^+ ^3.9 mEq/L

Coagulation profile	Platelet count 2.76 lakh/mm^3^, PT 25 (patient) and 14 (control) with a INR 1.8, APTT 31 (patient) and 28 (control)

We advised the patient to discontinue warfarin for four days and started on unfractionated heparin 4000 IU subcutaneously 6^th ^hourly. On the day of surgery, the last dose of heparin was administered 6 h prior to surgical incision and the coagulation studies prior to starting the surgery revealed PT 16 (patient) and 14 (control) with a INR 1.1, APTT 32 (patient) and 28 (control). Two units of packed red blood cells were cross matched in view of anemia. Fresh frozen plasma and protamine were reserved for emergency use in case of undue blood loss during the surgery. Her morning dose of insulin was skipped and she was kept fasting as per the standard guidelines. She received ampicillin 1.5 g and gentamicin 80 mg intravenously as infective endocarditis prophylaxis 30 minutes prior to skin incision.

As the patient was not willing for a regional anesthetic, general anesthesia was planned. Anesthesia was maintained intraoperatively with intravenous infusion of fentanyl 1 μg per minute and isoflurane in a mixture of oxygen and nitrous oxide. Neuromuscular blockade was maintained with vecuronium. The process of cementing was uneventful. The operation resulted in 300 ml blood loss which was replaced with one unit of packed red blood cells and crystalloids. Anesthesia was smooth and uneventful. After tracheal extubation, the patient was shifted to high dependency unit for monitoring.

Six hours from the last dose of infective endocarditis administration, the second dose of ampicillin 1.5 g was administered in the postoperative period to the patient. Four hours after the surgery, as the drains did not show any undue bleeding, it was decided to administer her next dose of unfractionated heparin. From next day morning, she was also started on warfarin and PT and APTT were repeated daily. By fourth day the INR was 3.0, heparin was stopped and warfarin continued as before and an ECHO was done which revealed normal functioning valves with no clot or vegetation. Now, ten days after the procedure, she is ready to be discharged home.

## Discussion

Patients with mechanical prosthetic heart valves are exposed to a significant threat of thromboembolism and valve dysfunction if proper anticoagulation is not achieved. Patients with new generation prosthetic mechanical mitral valves should receive warfarin to a target INR of 2.5–3.5 and for older types of valves the target INR should be 3.5–4.5 [1]. However, they may later be scheduled for elective or emergency non-cardiac procedures. This will be a tricky situation for the anesthesiologist to face as discontinuation of anticoagulation in the perioperative period can precipitate life threatening thromboembolism whereas continuation may cause significant bleeding during surgery. The risk of thromboembolism by withholding warfarin in patients with mechanical valve prostheses with atrial fibrillation is found to vary between 1% to 20% in various studies [2-6]. The prosthetic valve itself may get occluded by thrombus in 1–13% cases [7]. There are no large randomized studies that compare different perioperative anticoagulant regimens for patients with mechanical heart valves undergoing surgery. Because of the high risk of hemorrhage, the INR should be within the normal range before the procedure. In patients with heart valves, both the European Society of Cardiology and the Fourth American College of Chest Physicians Consensus Conference on Antithrombotic Therapy have recommended perioperative heparinization to minimize the risk of thrombosis resulting from the return to a normal INR [8,9].

Oral anticoagulation should be discontinued atleast three days before any major surgery and as a compromise between no anticoagulation and intravenous heparin, subcutaneous conventional heparin or low-molecular-weight heparin in prophylactic doses is substituted preoperatively. A dose of heparin should be given 3–6 h preoperatively in patients at high risk of thromboembolic events and heparin should be restarted as soon as possible post operatively (preferably within 12 h). Warfarin is restarted 24 hours postoperatively or when patients can start oral intake. Heparin should be continued till the INR is in the therapeutic range for at least 48 h, to enable a reduction in all the vitamin-K-dependent clotting factors [10,11].

In emergency surgery, the affect of warfarin needs to be neutralized by FFP, the dose of which depends upon the individual and this is titrated till INR < 1.5. In addition to this, vitamin K may also be given intravenously in small doses as large doses may lead to resistance to warfarin when it is restarted following surgery.

## Conclusion

We conclude that in patients with mechanical prosthetic heart valves for major elective surgery like bipolar hemireplacement arthroplasty, warfarin should be changed to unfractionated heparin atleast 4–5 days prior to surgery. INR should be less than 1.5 for proceeding with surgery. In view of risk of possible thrombosis, unfractionated heparin should be discontinued 3–6 h prior to surgery and restarted within 6–12 h of completion of the operation. Infective endocarditis prophylaxis should be administered to all patients with prosthetic heart valves.

## Competing interest

The authors declare that they have no competing interests.

## Authors' contributions

All of the authors were involved in the management of the case and finalizing the article. All of the authors were involved in the process of editing, correcting, and finalizing the manuscript. All authors have read and approved the final manuscript.

## Consent

The patient and the patient's relatives are pleased to give written consent for publishing this particular case scenario.
